# Back to the roots: Fine‐scale insights into adaptive foraging by a high‐Arctic large herbivore

**DOI:** 10.1002/ecy.70574

**Published:** 2026-10-23

**Authors:** Samantha P. H. Dwinnell, Emma Djurberg, Oline Eikeland, Leif Egil Loe, Steve D. Albon, Larissa T. Beumer, Åshild Ø. Pedersen, Stefaniya Kamenova, René van der Wal

**Affiliations:** ^1^ The University Centre in Svalbard Longyearbyen Norway; ^2^ Norwegian University of Life Sciences Ås Norway; ^3^ Umeå University Umeå Sweden; ^4^ UiT – The Arctic University of Norway Tromsø Norway; ^5^ The James Hutton Institute Aberdeen UK; ^6^ Norwegian Polar Institute Tromsø Norway; ^7^ Centre for Ecological and Evolutionary Synthesis University of Oslo Oslo Norway; ^8^ National Museum of Natural History, Bulgarian Academy of Sciences Sofia Bulgaria; ^9^ Swedish University of Agricultural Sciences Uppsala Sweden

**Keywords:** adaptive foraging, climate change, food resources, roots, Svalbard reindeer, vegetation strata

A clever herbivore should forage selectively in a way that maximizes energetic returns (Owen‐Smith & Novellie, 1982). The nutritional benefit of each bite, determined by the energy and nutrients gained, varies across space and time (Hobbs et al., 2003) as seasonally shifting environmental conditions drive plant phenology and quality (Klein, 1990). There is a growing abundance of research showing phenology tracking of food resources at their most nutritious state at the landscape level through use of remote‐sensed technologies (Abrahms et al., 2021), but underlying bite‐scale processes of forage selection in wild, free‐ranging large herbivores remain difficult to reveal.

Foraging behavior at the bite level underlies movement patterns at the landscape level, and herbivores seasonally tracking plants at their optimal phenological stages, even at finer scales, should be an adaptive foraging strategy (Owen‐Smith et al., 2010), driven by seasonally predictable shifts in energy and nutrient availability (Abrahms et al., 2021). Climate change, however, is disrupting predictability of food resources, with more extreme weather shifting plant availability and phenology in space and time, especially in the Arctic (Walsh et al., 2020). Earlier studies have emphasized the fitness costs of mismatch between plant phenology in spring and timing of parturition (Post & Forchhammer, 2008), but the misalignment between food availability and energetic demands of large herbivores during autumn is understudied (Mallory & Boyce, 2018). Considering the importance of late‐season forage for recovery of female body stores (Gustine et al., 2017), the ability to adjust foraging strategies to capitalize on variable conditions across seasons is likely to impact fitness (Wong & Candolin, 2014). Given the rapid pace of environmental change driven by Arctic warming (Rantanen et al., 2022), detection of flexibility in foraging strategies, across scales, is needed to determine the adaptive capacity of herbivore species and hence their population resilience (Beever et al., 2017).

To understand how flexible foraging strategies may be to shifting conditions, we examined how Svalbard reindeer track food resources, at the bite level, within the fastest‐warming region on Earth (Isaksen et al., 2022). We focused on late summer and autumn, a period characterized by generally accessible food but declining nutritional quality. In 2022 and 2023, we recorded forage selection across 26 individually marked female reindeer (of which 13 were sampled in both years) in Nordenskiöld Land, Svalbard (78° N, 15° E). Between August and October, we recorded the species and phenological stages of plants eaten by each reindeer at their exact foraging patch (10 × 10 cm sample area), while matching fecal samples from the same individuals, during the same time of the foraging observation, were collected and frozen for DNA metabarcoding diet analysis. An observer using a spotting scope (mean distance 331 m; SD 160 m) identified exact foraging patches from a distance, directing other team members with VHF radios to the precise feeding location (this method was independently tested and verified to a 10‐cm accuracy in site relocation up to 600‐m distance), with feeding identified by bite marks. We observed that contrasting autumn weather conditions between years resulted in substantial differences in the overall foraging strategies. In 2022, the ground froze (i.e., ground temperatures below 0°C for more than 1 week; measured from 128 iButtons placed at the ground surface across an elevational gradient throughout the study area) nearly 1 month later compared to 2023 (Figure 1a). Unexpectedly, this allowed reindeer to dig for belowground vegetation (e.g., roots of dwarf shrubs) until October 11, while in 2023, this resource was effectively inaccessible after September 17. Instead, in 2023, ground freezing occurred before complete senescence, “snap freezing” the aboveground green vegetation. Reindeer responded to this contrasting autumn condition by shifting foraging from belowground to aboveground vegetation strata (Figure 1), presumably tracking the most nutritious plant parts. During the warm autumn of 2022 (Figure 1a; September mean temperature 3.2 ± 0.5°C and just 4 days below 0°C), the aboveground vegetation had completely senesced with little green tissue available (Figure 1b). Reindeer fed on belowground vegetation (i.e., roots), especially in October (Figure 1d). Roots eaten were largely of the dwarf shrub, *Salix polaris* (60%). Conversely, during the colder autumn in 2023 (Figure 1a; September mean temperature 0.5 ± 0.7°C and 14 days below 0°C), reindeer fed on aboveground, frozen‐green vegetation (Figure 1c,e) that were mostly grasses (Poaceae, Juncaceae, and Cyperaceae; 68%) and forbs (including *Stellaria* spp. and *Cerastium* spp.; 29%). Diet composition from DNA metabarcoding of feces aligned with general patterns from direct observation (Figure 2) and revealed increased consumption of shrubs in the warmer autumn of 2022 (Figure 2a,b), including observed root eating (Figure 2c), while more grasses and forbs were consumed in the colder autumn of 2023 (Figure 2d,e), including observed eating of snap‐frozen green foliage (Figure 2f). Long‐term trends in autumn temperatures show an increase in mean September temperatures 1998–2023 (β = 0.07; *p* = 0.04; Appendix S1: Figure S1), with the warm autumn of 2022 being typical of the new climate regime.

**FIGURE 1:**
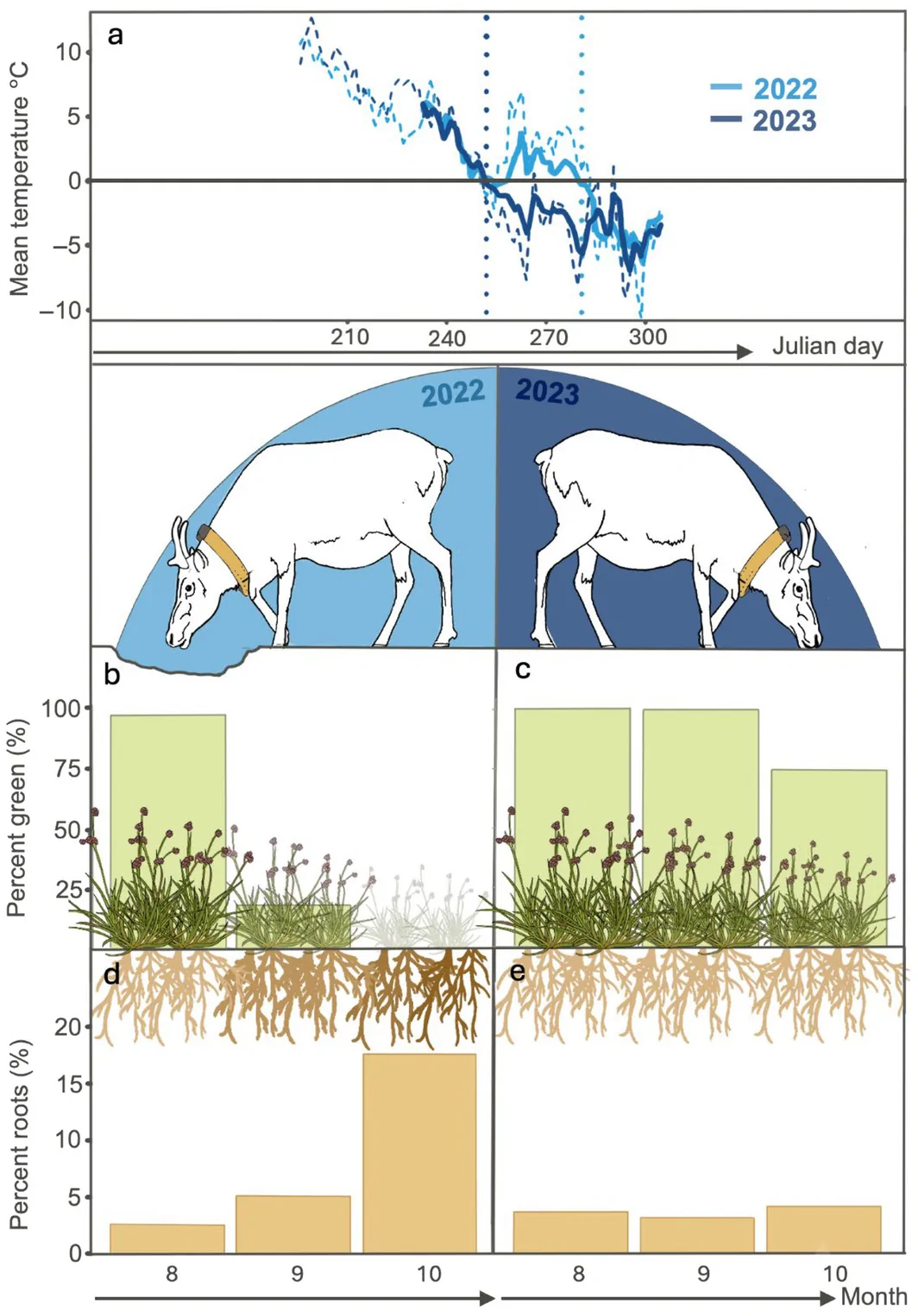
(a) Mean daily air (dashed) and ground (solid) temperatures from July 1 to November 1 in 2022 (light blue) and 2023 (dark blue). Vertical dotted lines are the first day of autumn when temperatures were below 0°C for at least one week. (b and c) Average monthly percent (%) of green vegetation (green bars) and (d and e) average monthly percent (%) of roots (orange bars) eaten by reindeer in a foraging patch between August–October. Data were obtained by direct observation in 2022 and 2023 in Nordenskiöld Land, Svalbard. Original illustrations created by Emma Djurberg.

**FIGURE 2:**
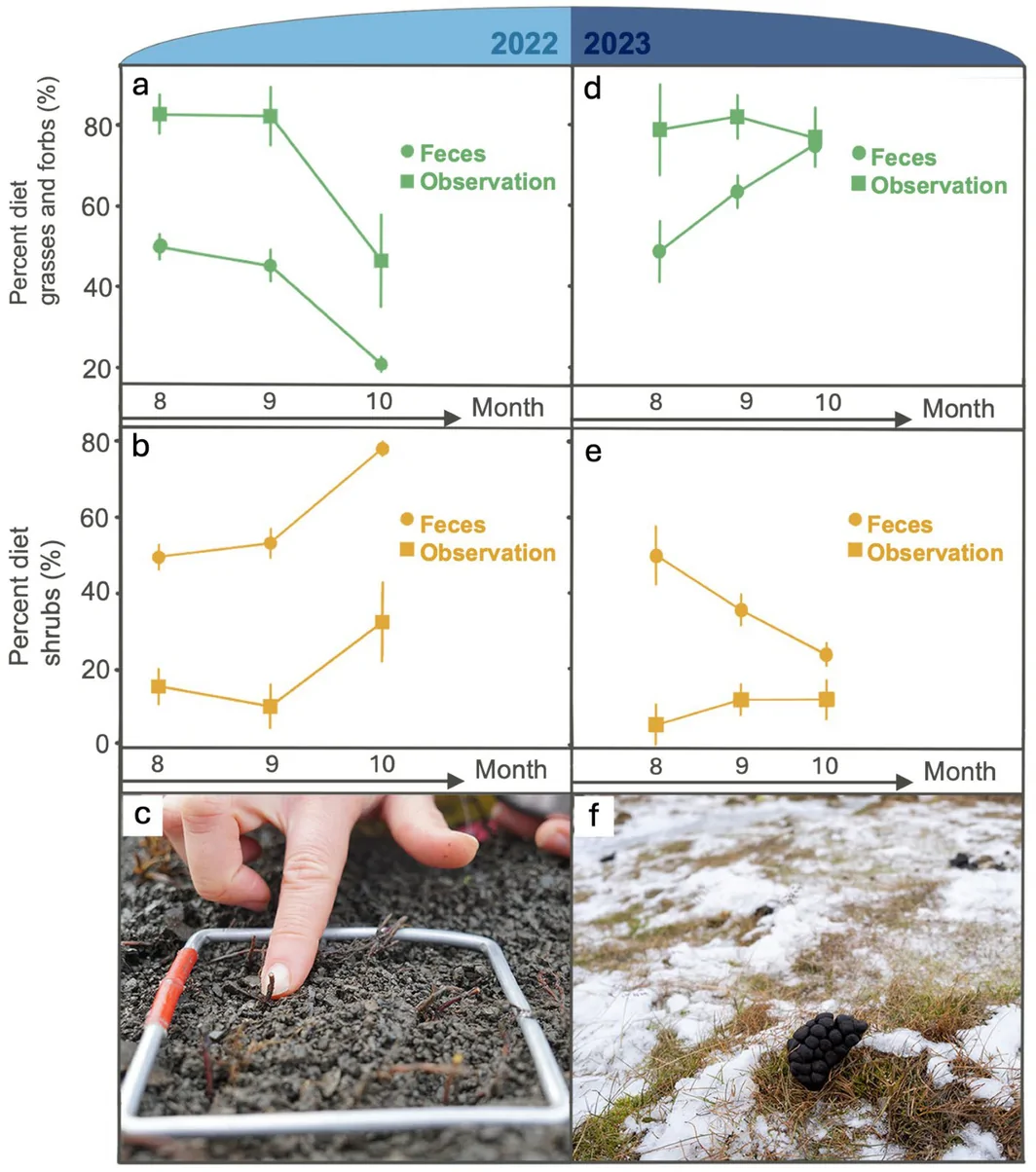
Monthly mean percent (%) of the diet (shown with SE) comprised of (a and d) grasses and forbs (green) and (b and e) shrubs (orange) in 2022 and 2023. Data were obtained by direct observation of grazed plants (squares) and DNA metabarcoding of fecal samples (circles). Photos taken in Nordenskiöld Land, Svalbard, of (c) *Salix polaris* roots eaten within an observation plot (taken on September 30, 2022), and (f) frozen green grass around a fecal sample (taken on October 2, 2023). Photo images (c and f) by Samantha P. H. Dwinnell.

Our findings of contrasting foraging behaviors, and especially the shift to root selection of a shrub, in response to contrasting environmental conditions at the onset of winter, demonstrate a new aspect of foraging plasticity in the Svalbard reindeer. This highlights how the selection of resources (e.g., grasses or shrubs), or vegetation strata (e.g., aboveground or belowground), used by large herbivores may not always be the most optimal resource under all environmental conditions. Evidence of free‐ranging herbivores tracking phenological variation in resources is largely based on aboveground food resources that vary at broad spatial scales (e.g., across landscapes; Abrahms et al., 2021). Here, we show that Svalbard reindeer select food resources at the finest scale—parts within an individual plant—including belowground roots when available in warmer autumns. At the end of summer, sugars and proteins are transported to roots for storage, shifting available nutrients from aboveground to belowground (Chapin et al., 1980), restricting the accessibility of nutrients for grazing large herbivores. Root biomass of Arctic plants is about 10 times greater than aboveground biomass across habitats (Anderson et al., 2012), so accessible roots in autumn may provide a treasure trove of nutritional food resources for Svalbard reindeer. Fitness‐related benefits from warmer autumns have been shown for Svalbard reindeer, related to delay in the onset of winter (Loe et al., 2021), and our findings show that such a delay provides access to belowground resources, a condition which may be of growing importance for reindeer under climate change (Dwinnell et al., 2024), especially considering our observed trends of warming autumns. Furthermore, a higher proportion of the dwarf shrub, *S. polaris*, in the Svalbard reindeer's autumn diet—likely the roots we observed to be most consumed here—has been shown to have a positive effect on body mass, with potential benefits for fitness (Kamenova et al., 2025). Although it remains unknown whether aboveground vegetation snap‐frozen before plant senescence in cold autumns is nutritionally better for reindeer than feeding on belowground roots in warmer autumns, it is likely that a diversity of foraging strategies promotes population resilience under increasingly variable environmental conditions (Schindler et al., 2010). Moving forward, the role of belowground roots as a source of food for Arctic large herbivores needs to be considered when evaluating availability and quality of food resources in tundra ecosystems.

Assessment of large herbivore diet and food quality is largely based on proxies that provide a broad understanding about diet composition. This often leaves the remaining unknown of “what do animals *actually* eat.” Although DNA metabarcoding or microhistology of fecal or rumen samples (e.g., see Bjørkvoll et al., 2009 for Svalbard reindeer) have become standard for diet evaluation, these methods fail to discern between the different plant parts consumed, leading to assumptions of exclusive aboveground foraging in large herbivores. Assessment of herbivore diets from direct field observations is expected to differ from DNA metabarcoding due to differences in the temporal scale of observations (e.g., instantaneous with direct observations vs. integrated across several days with fecal‐based proxies; Kamenova et al., 2024), and due to detection biases inherent in each method (e.g., human error with direct observation, and varying detection probabilities among plant species and environmental contaminations with proxies; Ando et al., 2020). Nevertheless, switching consumption of shrubs with grasses and forbs between the two contrasting autumns was detected with both methods, but with the advantage of direct observations revealing the discovery of feeding on shrub roots during the warmer autumn. This was only possible through extensive time spent in the field and highlights the ongoing need for “good‐old‐fashioned” field observations for uncovering intricate behaviors. To advance the field of resource tracking in wild animals, we need to couple methods for detecting forage selection across scales, including ongoing, direct observations that capture the array of foraging strategies that may help them adapt to rapid environmental change.

In terrestrial ecosystems, the impact of climate warming in the autumn, and delay in the onset of winter, on life history events has been less well studied than the consequences of earlier springs (Gallinat et al., 2015). Despite evidence of plant phenology in autumn having greater influence on timing of parturition than onset of spring (Kourkgy et al., 2016), phenological mismatches in autumn remain largely overlooked in large herbivores (Gustine et al., 2017). It is possible that the nutritional consequences of drastic shifts in plant phenology at the end of the growing season, as observed here, could have similar or greater effects on fitness, but flexible foraging strategies could mitigate such consequences. Our work reinforces the needed shift in focus to understanding how climate‐driven shifts in autumn plant phenology and nutrition can impact large herbivores' life histories and population growth.

## AUTHOR CONTRIBUTIONS

Samantha P. H. Dwinnell, René van der Wal, Leif Egil Loe, Oline Eikeland, and Emma Djurberg developed and designed the study. Leif Egil Loe, Steve D. Albon, René van der Wal, and Åshild Ø. Pedersen acquired funding. Samantha P. H. Dwinnell, Oline Eikeland, and Emma Djurberg collected the data. Stefaniya Kamenova conducted DNA metabarcoding diet analysis. Samantha P. H. Dwinnell performed the analysis. Emma Djurberg created illustrations and designed figures. All coauthors contributed to revisions of the manuscript before submission.

## CONFLICT OF INTEREST STATEMENT

The authors declare no conflicts of interest.

## Supporting information


Appendix S1.

## Data Availability

All data (Dwinnell et al., 2026), including iButton, foraging patch observation, and DNA metabarcoding of feces, are available in Dryad at https://doi.org/10.5061/dryad.vhhmgqp9t.
