# Supplementary material for: Back to the roots: Fine‐scale insights into adaptive foraging by a high‐Arctic large herbivore
Source: Ecology. 2026 Oct 23;107(10):e70574. doi: 10.1002/ecy.70574 (PMC13694197; doi:10.1002/ecy.70574)
Supplement: Supplementary file 1 [file ECY-107-e70574-s001.pdf]

Appendix S1

Back to the roots: Fine-scale insights into adaptive foraging by a high-Arctic large herbivore

Samantha P. H. Dwinnell, Emma Djurberg, Oline Eikeland, Leif Egil Loe, Steve D. Albon, Larissa T. Beumer, Åshild Ø. Pedersen, Stefaniya Kamenova and René van der Wal

Ecology

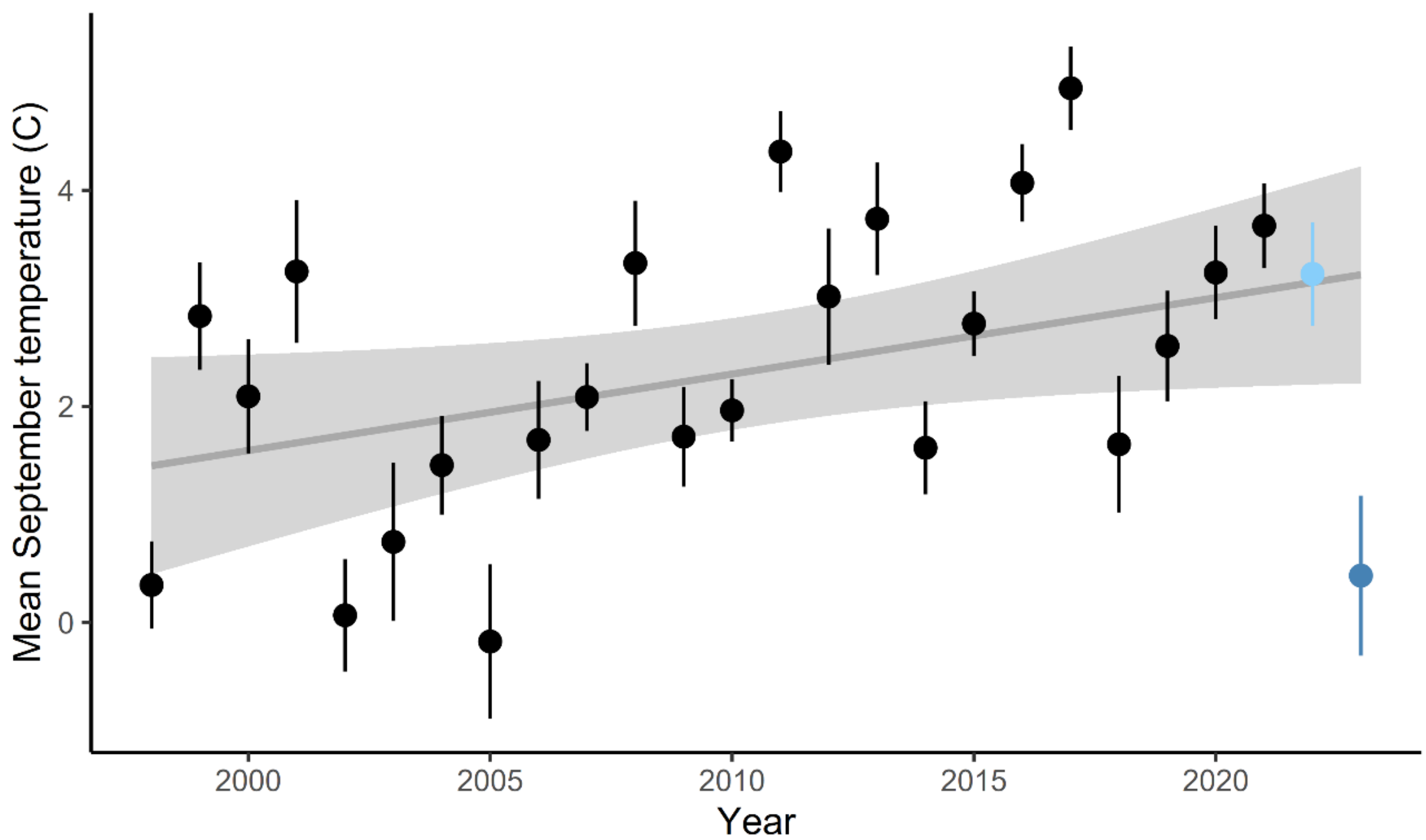

Figure S1. Means and standard deviations of daily September temperature (°C) measured at Svalbard Airport, Longyearbyen, Svalbard 1998-2023 (station number SN99840; the Norwegian Centre for Climate Services; https://seklima.met.no/), including a fitted linear trend with 95% confidence band of change over time. Mean September temperatures are highlighted for 2022 (light blue) and 2023 (dark blue).
